# Supplementary material for: Synergistic Effect of Y Doping and Reduction of TiO2 on the Improvement of Photocatalytic Performance
Source: Nanomaterials (Basel). 2023 Aug 7;13(15):2266. doi: 10.3390/nano13152266 (PMC10420816; doi:10.3390/nano13152266)
Supplement: Supplementary file 1 [file nanomaterials-13-02266-s001.zip › nanomaterials-2508340-supplementary.pdf]

Synergistic Effect of Y Doping and Reduction of TiO_2 on the Improvement of Photocatalytic Performance

Xijuan Li [1], Hongjuan Zheng [2,*], Yulong Wang [3], Xia Li [1,2], Jinsong Liu [1], Kang Yan [2], Jing Wang [2] and Kongjun Zhu [2,*]

[1] College of Materials Science and Technology, Nanjing University of Aeronautics and Astronautics,
Nanjing 210016, China; lxjnuaa@163.com (X.L.); lixia170904@nuaa.edu.cn (X.L.); jsliu@nuaa.edu.cn (J.L.)

[2] State Key Laboratory of Mechanics and Control for Aerospace Structures, Nanjing University of Aeronautics and Astronautics, Nanjing 210016, China; yankang@nuaa.edu.cn (K.Y.); wang-jing@nuaa.edu.cn (J.W.)

[3] Department of Applied Physics, The Hong Kong Polytechnic University, Hong Kong 999077, China; yulong1.wang@polyu.edu.hk

* Correspondence: zhenghj2012@126.com (H.Z.); kjzhu@nuaa.edu.cn (K.Z.)

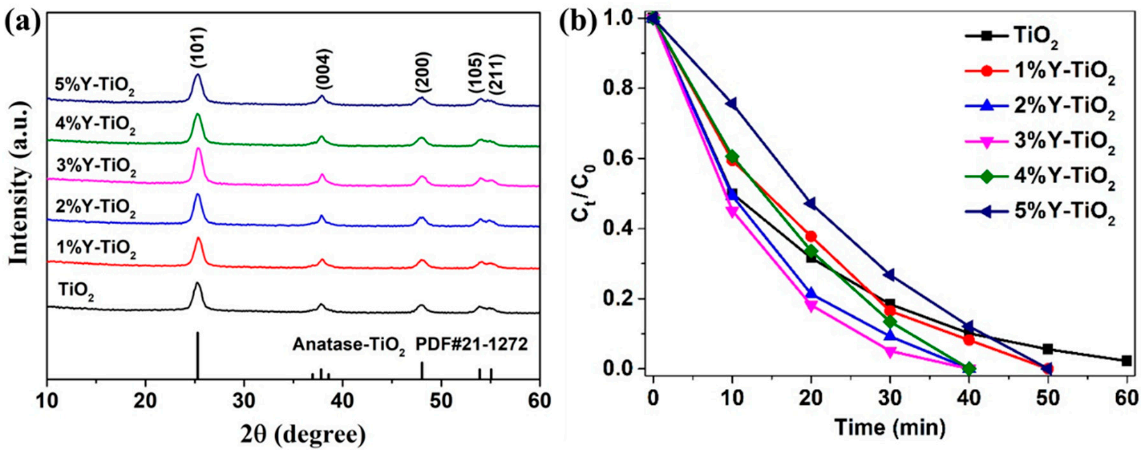

Figure S1. XRD patterns(a) and degradation curves (b) of pure TiO_2 and various Y-doped TiO_2 (1~5%) samples.

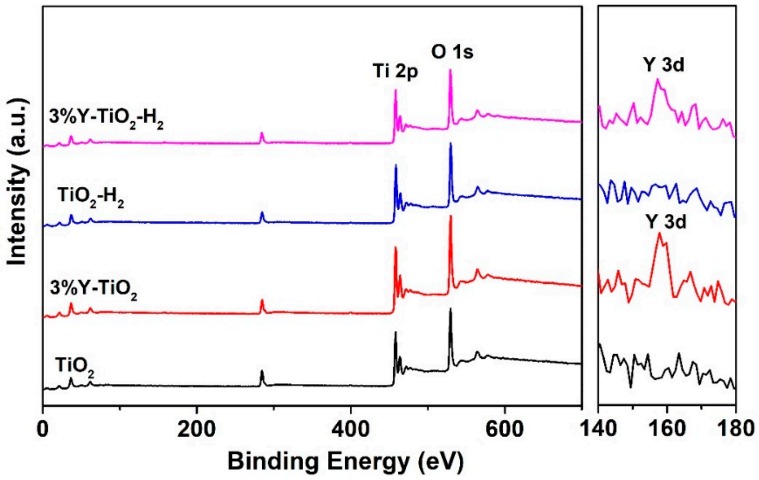

Figure S2. Survey scanning XPS spectra of TiO_2, 3% Y-TiO_2, TiO_2-H_2 and 3% Y-TiO_2-H_2 samples.

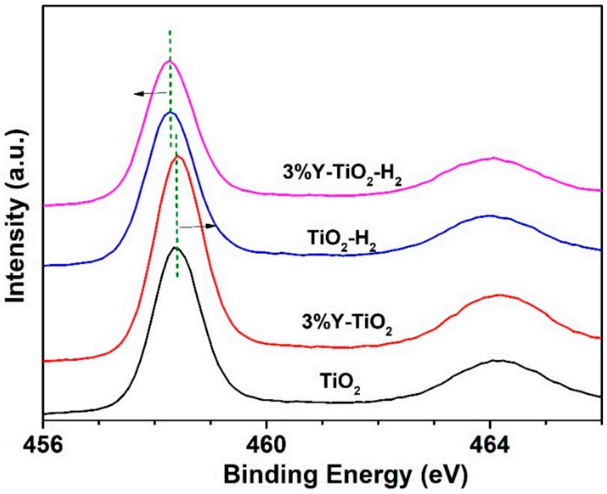

Figure S3. XPS spectra of Ti 2p for TiO_2, 3%Y-TiO_2, TiO_2-H_2 and 3%Y-TiO_2-H_2 samples.
